# Impact of nurses’ health beliefs about caring for older adults on their own preparation for old age

**DOI:** 10.3389/fpubh.2025.1609198

**Published:** 2025-08-19

**Authors:** Se Jin Hwang, Minkyung Gu, Saekyae Shin, Sohyune Sok

**Affiliations:** ^1^Department of Nursing, Graduate School, Kyung Hee University, Seoul, Republic of Korea; ^2^Department of Nursing, College of Health Science, Daejin University, Pocheon-si, Republic of Korea

**Keywords:** old age, preparation, health belief, retirement, nurse

## Abstract

**Background:**

The older adults is increasing worldwide, and South Korea in particular is experiencing a rapid increase in the older adults. In this situation, related research targeting nurses who care for the older adult is necessary.

**Objective:**

This study was to examine the relationships among preparation for old age and health beliefs, and the factors influencing preparation for old age of young nurses in their 20s and 30s who care for older adults in general hospitals.

**Methods:**

A cross-sectional descriptive design was employed. Participants were 110 nurses working in general hospitals, South Korea. Measures were the general characteristics list, the preparation scale for old age, and health belief scale.

**Results:**

Level of preparation for retirement (*β* = −0.53, *p* < 0.001), department (*β* = 0.34, *p* < 0.001), health beliefs (β = 0.32, *p* < 0.001), desired retirement age (β = 0.19, *p* = 0.022), annual salary (β = −0.18, *p* = 0.048), and chronic disease (β = 0.17, *p* = 0.028) were all statistically significant variables in the Step-2 regression model. Explanatory power was 69.2%.

**Conclusion:**

Improving preparation for retirement and health beliefs could promote preparation for old age. Department, desired retirement age, annual salary, and chronic disease need to be considered when developing and implementing interventions for improving preparation for old age. Nursing managers or nurses need pay attention to factors influencing preparation for old age of young nurses caring for older adult patients in general hospitals.

## Introduction

South Korea, a rapidly aging OECD nation, sees its older population projected to exceed 46.4% by 2070 (1, 2). This demographic shift raises living costs and heightens the need for early retirement planning (3, 4). Young people in their 20s and 30s, crucial for preparing a super-aged society, often lack readiness, as shown by a 2022 Statistics Korea Retirement Readiness Survey (5–8). Effective old-age preparation counters potential health, financial, and social challenges. With issues like pension depletion looming, young individuals proactively need to plan for secure, fulfilling later years, independent of limited social security (9–11).

It is worth noting that nurses can directly participate in the care of old adults, both in terms of their physical health and their social and psychological life after retirement. Through this experience, nurses can reflect thoroughly on the success of their future life after retirement (12, 13). In addition, while caring for sick old adults, nurses may indirectly experience changes in their perception of life after retirement and reflect on their health beliefs related to their illness (14, 15). Health beliefs are known to be crucial predictors of an individual’s health-related behavior. They are firm beliefs that individuals are vulnerable to illness and that the occurrence of the disease is serious (16). In other words, if they perceive that there are numerous benefits to their health beliefs and fewer obstacles, they are more likely to engage in health-promoting behaviors. In this approach, health beliefs help people anticipate negative situations and pursue a healthy life by taking positive and proactive behaviors when they think their health may be threatened or when they are concerned that they will get sick (17). Health beliefs are actually a significant predictor of an individual’s health-related behaviors and can significantly influence preparation for old age. These beliefs include perceptions of vulnerability to disease and the severity of disease occurrence. The more benefits and fewer perceived barriers to health beliefs an individual perceives, the more likely they are to engage in health-promoting behaviors. Therefore, health beliefs anticipate negative situations and encourage positive behaviors to maintain a healthy lifestyle, which is essential for planning for a stable old age. Preparation for old age involves proactive planning and actions to handle potential challenges in later life, such as illnesses, poverty, and loneliness. It includes detailed planning for physical, economic, social, and psychological concerns that may arise during old age (17). These health beliefs are grounded in the Theory of Planned Behavior (TPB) and the life course perspective. The Theory of Planned Behavior (TPB) and the life course perspective are frameworks used to understand human behavior and development over time. The Theory of Planned Behavior (TPB), developed by Ajzen (18), is a psychological theory that links beliefs and behaviors. TPB posits that an individual’s behavior is driven by behavioral intentions, which are shaped by three factors: attitude toward the behavior, subjective norms, and perceived behavioral control. TPB is often used to predict behavioral patterns based on an individual’s existing attitudes and intentions. The life course perspective (19) is used in sociology and public health to understand how age, relationships, general life transitions, and social change affect people’s lives from birth to death. This theory emphasizes the importance of time, context, and process in understanding people’s lives, structural contexts, and social change. The life course perspective examines how various stages of life are interconnected and how early experiences can influence later life. Both theories play an important role in understanding behavior and development, but TPB focuses more on immediate behavioral intentions, whereas the life course perspective provides a broader view of individual development across the lifespan.

Currently, the demand for specialized medical services for old adults is increasing, and nurses’ roles in this aging society are emphasized in various aspects. Nevertheless, since most nurses working in hospitals are in their 20s and 30s and are deemed young and relatively in good health, they may not consider old age planning based on their own health beliefs (12). Furthermore, nurses caring for old adults tend to neglect their own health due to unhealthy lifestyle habits and stress caused by the nature and intensity of shift work (13). Existing efforts and interventions to prepare for old age of healthcare professionals caring for the older adults have only provided discounts on medical and screening fees as a welfare service, and in many cases, hospitals or institutions rather encourage early retirement (12, 15). Indeed, nurses caring for old adults need to take a look on their health beliefs and consider whether they are adequately preparing for old age.

However, there have been no prior studies on the health beliefs and preparation for old age of young nurses in their 20s and 30s caring for old adults. Although there have been studies related to preparation for old age in various occupations (20–23), very few addressed the preparation for old age with respect to the health beliefs of young nurses in their 20s and 30s who are currently in charge of caring for old adults in the health care field. At this point, nurses need to exercise their subjective health beliefs about their health and to explore in detail how they prepare and plan for old age. This study aims to provide fundamental data for developing positive measures to prepare for a desirable life after retirement by identifying the current awareness of their health beliefs among young nurses in their 20s and 30s caring for old adults.

The purpose of this study was to examine the influence on the preparation for old age of young nurses in their 20s and 30s who care for older adults in general hospitals. The aims were to (a) identify the general and job-related characteristics of study participants; (b) examine the levels of the preparation for old age and health belief of study participants; (c) examine the differences on the preparation for old age and health belief according to the general and job-related characteristics of study participants; (d) examine the correlation between the preparation for old age and health belief of study participants; (e) examine the factors influencing the level of preparation for old age of study participants.

## Methods

### Study population

A cross-sectional descriptive design was employed. Study participants were 110 nurses working in general hospitals, South Korea. They were selected using convenience sampling. Inclusion criteria were nurses in their 20s and 30s who had worked in the hospital for more than 3 months, were currently caring for old adults, and understood the purpose of this study. Not included criteria were nurse managers, and new nurses who had worked for less than 3 months based on the reliability of this study because it was judged difficult to provide direct care for old adults.

The sample—110 people—was acquired using the G*Power 3.1.9 program, with a significance level (⍺) of 0.05, a power (1-*β*) of 0.80, and an effect size of 0.15 (24). Considering that a 10% dropout rate would occur, 123 questionnaires were disseminated. Then, 110 questionnaires were collected (89.43% collection rate) and included in the final data.

### Measurements

The measurement tools used in this study were validated for content validity and reliability by two nursing professors, one geriatric medicine professor, and two clinical nurses. Statistical techniques were used to verify the reliability of the measurement tools.

### General and job-related characteristics of study participants

A set of general characteristic variables consisted of a total of 12 items including gender, age, marital status, education, religion, total clinical experience, department, current position, annual salary, desired retirement age, preparation for retirement, and chronic disease.

### Preparation for old age

To assess preparation for old age, it was an adopted tool from Kang and Yeom (25) who aimed to help nurses pursue a stable life after retirement. This instrument comprises 28 questions, with nine for economic readiness, 10 for physical readiness, and nine for emotional readiness, such as “I save every month for financial deposit for old age,” “I train my body through exercise for a healthy life,” and “I try not to accumulate stress.” This instrument uses a five-point Likert scale, with values ranging from a minimum of 28 to a maximum of 140 points, with higher scores indicating greater preparation for old age. As regarding the preparation scale for old age, in Kang and Yeom’s study (25), the reliability of the instrument was Cronbach’s *α* = 0.90, but in this study, it was Cronbach’s α = 0.87.

### Health belief

To assess health belief, it was a modified tool from Lee and Jung (26). The four-subarea instrument includes 22 questions—four about perceived sensitivity, five about perceived severity, six about perceived benefit, and seven about perceived disability. The questions include statements such as “The thought that a chronic disease can occur or worsen frightens me” and “If I manage stress well, I will prevent the occurrence or worsening of chronic diseases.” This instrument uses a five-point Likert scale, with values ranging from a minimum of 22 to a maximum of 110 points. The greater the value of the instrument, the higher the health belief. As for the health belief scale, in Lee and Jung’s study (26), the reliability of the instrument was Cronbach’s *α* = 0.80, while in this study, it was Cronbach’s α = 0.87. All of the above tools were verified for content validity by two nursing professors.

### Data collection

This study was conducted after receiving approval from the K University Bioethics Review Committee. Prior to data collection, the researcher visited the general hospital in person, asked for cooperation from the nursing department and head nurses, and explained the purpose and content of this study directly. In addition, all nurses were informed in advance of the confidentiality of personal information provision and anonymity. Data collection was conducted from October to November, 2023, and a questionnaire was administered to nurses currently caring for old adults at a general hospital. It took about 30 min for each nurse to complete the questionnaire, and the researcher collected the completed questionnaire after placing it in an envelope and taping it.

### Ethical considerations

This study was approved by K University Institutional Review Board (IRB No. KHSIRB-23-471, Approval date October 24, 2023). This study explained the purpose and procedure of the study to nurses who work at general hospitals and currently care for old adults. Afterwards, they were asked to cooperate with the data collection and were told that it would not be used for any purpose other than the study. In addition, they were told that there would be no disadvantages for not participating in the study, and the researcher distributed a questionnaire to those who agreed to participate in the study and had them fill it out themselves. The questionnaire was stored in the researcher’s locker with a locker so that no one other than the researcher could access it, and it was stored for 3 years after the end of the study, after which all documents were permanently shredded.

### Data analysis

In this study, the collected data were analyzed using the SPSS/WIN 29.0 Program (IBM Corp., Armonk, NY, United States). Descriptive statistics and frequency analysis were used to confirm the general and job-related characteristics of study participants. The level of preparation for old age and health beliefs of study participants were analyzed using the mean and standard deviation. In order to examine the differences in the level of preparedness for old age and health beliefs according to the general and job-related characteristics of study participants, the independent *t*-test, ANOVA, and Scheffe *post-hoc* test was used. The correlation between the level of preparation for old age and health beliefs of study participants was analyzed using Pearson’s coefficient correlation. In order to examine the factors influencing the level of preparation for old age of study participants, hierarchical stepwise multiple regression was used for analysis.

## Results

### General and job-related characteristics of nurses caring for old adults

This study covered 110 nurses who cared for old adults, with five males (4.5%) and 105 females (95.5%). In terms of age, 55 nurses (50.0%) were aged 25–29, followed by 30 nurses (27.3%) aged 30 to 34, with an average age of 29.85 years. The majority of nurses caring for old adults were unmarried 82 nurses (74.5%), and the majority had a college degree (80.9%). Sixty-four nurses (58.2%) had no religious affiliation, whereas 98 nurses (89.1%) had less than 10 years of total clinical experience. The internal medicine ward had the most nurses, totaling 44 (40.0%) among the departments they worked in. Then, general nurses accounted for the majority with 107 (97.3%). Next, 56 nurses (50.9%) aged 50–59 years responded they would like to retire, while 47 nurses (42.7%) stated they were “just planning” to prepare for old age. In this study, 106 nurses (96.4%) caring for old adults were free of chronic diseases (Table 1).

**Table 1:** General and job-related characteristics of nurses caring for older adults.

Characteristics	N	%
Gender		
Male	5	4.5
Female	105	95.5
Age (year)		
25>	15	13.6
25–29	55	50.0
30–34	30	27.3
35–39	10	9.1
Mean ± SD	29.85 ± 3.93	
Marital status		
Yes	28	25.5
No	82	74.5
Education		
College	13	11.8
University	89	80.9
Attending graduate school	4	3.6
Finish graduate school	4	3.6
Religion		
Buddhism	10	9.1
Christian	25	22.7
Catholic	11	10.0
No	64	58.2
Total clinical experience (year)		
10>	98	89.1
Oct–14	8	7.3
15≤	4	3.6
Department		
Medical ward	44	40.0
Surgical ward	11	10.0
Intensive care unit	10	9.1
Others	45	40.9
Current position		
General nurse	107	97.3
Senior nurse	3	2.7
Annual salary (USD)		
20,300>	11	10.0
20,300–27,000	53	48.2
27,001–33,700	31	28.2
33,701≤	15	13.6
Desired retirement age (year)		
30–39	17	15.5
40–49	12	10.9
50–59	56	50.9
60≤	25	22.7
Preparation for retirement		
Actively preparing	4	3.6
Preparing to some extent	23	20.9
Just making plans	47	42.7
Not prepared at all	36	32.7
Chronic disease		
Yes	4	3.6
No	106	96.4

### Levels of preparation for old age and health beliefs of nurses caring for old adults

Nurses caring for old adults reported preparation for old age readiness scores ranging from 28 to 140, with a median of 84.00 and a mean of 85.41 (13.37) points. Economic readiness scores ranged from nine to 45, with a median of 27.00 and a mean of 24.97 (6.04), physical readiness scores from 10 to 50, with a median of 30.00 and a mean of 29.52 (6.09), and emotional readiness from nine to 45, with a median of 27.00 and a mean of 30.92 (4.73). Next, health beliefs scores ranged from 22 to 110, with a median of 66.00 and a mean of 76.85 (6.95). Perceived sensitivity, a subarea related to health beliefs, ranged from four to 20, with a median of 12.00 and a mean of 12.75 (3.55), perceived severity from five to 25, with a median of 15.00 and a mean of 15.68 (4.56), perceived benefit from six to 30, with a median of 18.00 and a mean of 26.39 (3.17), and perceived disability from 7 to 35, with a median of 21.00 and a mean of 22.03 (4.45). In this study, the mean values of nurses caring for old adults for preparation for old age and health beliefs were mostly similar to or higher than the median values. However, the mean values for economic readiness and physical readiness, which are subareas of preparation for old age, were slightly lower than the median values (Table 2).

**Table 2:** Levels of preparation for old age and health beliefs of nurses caring for old adults.

Variables	Possible score	Median	Mean ± SD	Variables	Possible score	Median	Mean ± SD
Preparation for old age	28–140	84.00	85.41 ± 13.37	Health beliefs	22–110	66.00	76.85 ± 6.95
Financial preparation	9–45	27.00	24.97 ± 6.04	Perceived sensitivity	4–20	12.00	12.75 ± 3.55
Physical preparation	10–50	30.00	29.52 ± 6.09	Perceived severity	5–25	15.00	15.68 ± 4.56
Emotional preparation	9–45	27.00	30.92 ± 4.73	Perceived benefit	6–30	18.00	26.39 ± 3.17
				Perceived disability	7–35	21.00	22.03 ± 4.45

### Differences on preparation for old age and health beliefs according to the general and job-related characteristics of nurses caring for old adults

The preparation for old age of nurses caring for old adults was a statistically significant difference in terms of total clinical experience (*F* = 1.58, *p* = 0.049) and level of preparation for retirement (*F* = 10.42, *p* < 0.001). The result from *post-hoc* test demonstrates that the level of preparation for old age of nurses with a total clinical experience of 15 years or more was higher than them of less than 10 years, or 10–14 years. The level of preparation for old age of nurses caring for old adults was also higher for nurses who claimed they were “actively preparing for retirement” and “preparing to some extent for retirement” compared to those who said they were “not preparing at all for retirement.” The level of health beliefs of nurses caring for old adults was a statistically significant difference in terms of their preparation for retirement (*F* = 0.76, *p* = 0.048). The result from *post-hoc* test exhibited that the health beliefs of nurses caring for old adults were higher for nurses who stated they were “preparing to some extent for retirement” compared to those who held they were “just planning for retirement” and “not preparing at all for retirement” (Table 3).

**Table 3:** Differences on preparation for old age and health beliefs according to the general and job-related characteristics of nurses caring for old adults.

Characteristics	Preparation for old age		Health beliefs	
	Mean ± SD	*t*-test or *F*- test (*P*) *Scheffe*	Mean ± SD	*t*-test or *F*- test (*P*) *Scheffe*
Gender				
Male	86.00 ± 4.00	−0.01 (0.093)	75.20 ± 3.63	−0.37 (0.192)
Female	86.04 ± 11.10		76.28 ± 6.51	
Age (year)				
25>	88.07 ± 11.17	0.54 (0.790)	77.13 ± 5.05	0.34 (0.882)
25–29	85.00 ± 11.21		76.55 ± 6.91	
30–34	86.03 ± 11.14		75.40 ± 6.71	
35–39	88.70 ± 7.80		75.60 ± 4.53	
Marital status				
Yes	86.43 ± 11.45	0.22 (0.709)	75.50 ± 7.24	
No	85.90 ± 10.73		76.48 ± 6.12	−0.70 (0.621)
Education				
College	85.31 ± 11.54	0.22 (0.913)	65.62 ± 5.98	0.18 (0.905)
University	86.31 ± 10.98		76.20 ± 6.60	
Attending graduate school	86.25 ± 12.84		78.25 ± 7.14	
Finish graduate school	82.00 ± 5.35		76.75 ± 3.77	
Religion				
Buddhism	82.70 ± 13.27	0.79 (0.320)	73.80 ± 7.77	2.11 (0.145)
Christian	86.20 ± 7.24		77.92 ± 7.13	
Catholic	89.91 ± 12.27		79.00 ± 4.43	
No	85.83 ± 11.44		75.47 ± 5.98	
Total clinical experience (year)				
10>	85.57 ± 10.90^a^	1.58 (0.049*) c > a,b	76.43 ± 6.47	0.57 (0.622)
10–14	87.13 ± 12.11^b^		75.25 ± 6.48	
15≤	95.25 ± 0.50^c^		73.25 ± 4.50	
Department				
Medical ward	83.09 ± 8.79	3.68 (0.212)	76.41 ± 6.02	2.25 (0.082)
Surgical ward	82.18 ± 12.79		76.36 ± 6.98	
Intensive care unit	85.90 ± 6.97		80.70 ± 5.38	
Others	89.90 ± 11.94		75.02 ± 6.60	
Current position				
General nurse	85.82 ± 10.89	−1.24 (0.415)	76.21 ± 6.35	−0.21 (0.298)
Senior nurse	93.70 ± 7.64		77.00 ± 9.85	
Annual salary (USD)				
20,300>	85.73 ± 10.75	0.03 (0.998)	74.64 ± 8.16	1.65 (0.344)
20,300–27,000	86.30 ± 10.87		77.36 ± 4.32	
27,001–33,700	85.62 ± 11.77		76.13 ± 7.49	
33,701≤	86.20 ± 10.02		73.60 ± 8.23	
Desired retirement age (year)				
30–39	84.29 ± 11.14	1.15 (0.337)	76.12 ± 6.00	0.13 (0.951)
40–49	81.50 ± 9.41		75.33 ± 6.91	
50–59	86.73 ± 11.27		76.54 ± 5.88	
60≤	87.84 ± 10.26		76.04 ± 7.78	
Preparation for retirement				
Actively preparing	94.00 ± 1.00^a^	10.42 (< 0.001*) a,b > d	74.50 ± 7.00^a^	0.76 (0.048*) b > c,d
Preparing to some extent	92.13 ± 8.70^b^		77.26 ± 5.68^b^	
Just making plans	87.49 ± 9.80^c^		75.32 ± 6.32^c^	
Not prepared at all	79.25 ± 10.51^d^		76.94 ± 6.93^d^	
Chronic disease				
Yes	81.50 ± 7.77	−0.85 (0.408)	82.00 ± 6.98	1.86 (0.735)
No	86.21 ± 11.00		76.01 ± 6.31	

*p < 0.05; a,b,c,d are the Scheffe test values in post hoc test.

### Correlations between preparation for old age and health beliefs of nurses caring for old adults

This study found a positive correlation between preparation for old age and the health beliefs of nurses caring for old adults (*r* = 0.32, *p* < 0.001). A positive correlation between perceived sensitivity (*r* = 0.24, *p* = 0.012) and perceived disability (*r* = 0.41, *p* < 0.001), which are subareas related to health beliefs, was also observed. In other words, the higher the preparation for old age of nurses caring for old adults, the greater their health beliefs. Specifically, the higher their preparation for old age, the greater their perceived sensitivity and perceived disability in terms of their health beliefs (Table 4).

**Table 4:** Correlations between preparation for old age and health beliefs of nurses caring for old adults.

Variables	Preparation for old age	Financial preparation	Physical preparation	Emotional preparation	Health beliefs	Perceived sensitivity	Perceived severity	Perceived benefit	Perceived disability
	r (*P*)								
Preparation for old age	1								
Financial preparation	0.76 (< 0.001*)	1							
Physical preparation	0.84 (< 0.001*)	0.41 (< 0.001*)	1						
Emotional preparation	0.77 (< 0.001*)	0.34 (< 0.001*)	0.57 (< 0.001*)	1					
Health beliefs	0.32 (< 0.001*)	0.14 (0.158)	0.33 (< 0.001*)	0.29 (0.002*)	1				
Perceived sensitivity	0.24 (0.012*)	0.03 (0.730)	0.31 (< 0.001*)	0.23 (0.017*)	0.28 (0.004*)	1			
Perceived severity	0.18 (0.061)	0.16 (0.098)	0.11 (0.265)	0.17 (0.082)	0.61 (< 0.001*)	0.13 (0.186)	1		
Perceived benefit	0.13 (0.192)	−0.09 (0.327)	0.18 (0.054)	0.24 (0.012*)	0.57 (< 0.001*)	−0.18 (0.054)	0.18 (0.063)	1	
Perceived disability	0.41 (< 0.001*)	0.14 (0.137)	0.53 (< 0.001*)	0.30 (0.002*)	0.32 (< 0.001*)	−0.37 (< 0.001*)	−0.31 (0.001*)	0.14 (0.145)	1

*P < 0.05.

### Impact on preparation for old age of nurses caring for old adults

The impact on the preparation for old age of nurses caring for old adults was analyzed by stepwise hierarchical multiple regression. As a result, the Step-1 regression model with general characteristics was statistically significant. Department (*β* = 0.29, *p* = 0.005) was the statistically significant variable in Step 1, and the explanatory power of the Step 1 regression model was 35.1%. In this research, Step 2 included the desired retirement age, the level of preparation for retirement, chronic diseases, and health beliefs, which were the main variables. Department (*β* = 0.34, *p* < 0.001), annual salary (β = −0.18, *p* = 0.048), desired retirement age (β = 0.19, *p* = 0.022), level of preparation for retirement (β = −0.53, *p* < 0.001), chronic disease (β = 0.17, *p* = 0.028), and health beliefs (β = 0.32, *p* < 0.001) were all statistically significant variables in the Step-2 regression model. Explanatory power increased by 34.1% compared to Step 1. The most impactful and important variable was the level of preparation for retirement, followed by department, health beliefs, desired retirement age, annual salary, and chronic disease. The explanatory power of the final regression model was 69.2% (Table 5).

**Table 5:** Impact on preparation for old age of nurses caring for old adults.

Variables	Model I					Model II				
	*B*	SE	*β*	*t*	*P*	*B*	SE	*β*	*t*	*P*
Gender	−0.69	5.13	−0.01	−0.13	0.893	−4.71	4.11	−0.09	−1.15	0.255
Age	−0.26	1.63	−0.02	−0.16	0.975	−2.50	1.34	−0.19	−1.87	0.065
Marital status	−0.39	2.65	−0.02	−0.15	0.884	−1.79	2.11	−0.07	−0.85	0.400
Education	0.24	1.93	0.01	0.13	0.901	1.17	1.54	0.06	0.76	0.447
Religion	0.84	1.00	0.08	0.84	0.401	1.56	0.79	0.15	1.97	0.052
Total clinical experience	3.02	2.83	0.12	1.07	0.287	0.47	2.37	0.02	0.20	0.843
Department	2.30	0.79	0.29	2.90	0.005*	2.68	0.63	0.34	4.25	< 0.001*
Current position	9.22	6.46	0.14	1.43	0.157	2.17	5.17	0.03	0.42	0.676
Annual salary	−1.05	1.41	−0.08	−0.75	0.457	−2.30	1.15	−0.18	−2.01	0.048*
Desired retirement age						2.11	0.91	0.19	2.33	0.022*
Preparation for retirement						−6.92	1.16	−0.53	−5.98	< 0.001*
Chronic disease						10.00	4.49	0.17	2.23	0.028*
Health beliefs						0.54	0.13	0.32	4.16	< 0.001*
Adj R2 = 0.351, *F* = 1.57, *p* = 0.014*						Adj R2 = 0.692, *F* = 6.79, *P* < 0.001*				

B, unstandardized coefficients; SE, standard error; ß, standardized coefficients; t, *t*-test; Adj. R^2^, adjust R-squared; *P < 0.05.

The assumptions of the regression analysis were tested and proved to be valid, and the assumptions of the regression equation were satisfied. First of all, the tolerance limit of multicollinearity was 0.53–0.92, which exceeded 0.10. The variance inflation factor (VIF) ranged from 1.07 to 1.89. Because the VIF value was not greater than 10, there was no issue of multicollinearity for all variables.

## Discussion

In South Korea, due to the aging population and the social culture that requires long-term employment even after retirement, there may be a sense of both the possibility of rest and the burden of having to find work again (8, 21). Given the lack of policies and systems for retirees, Korean cultural norms regarding retirement may be viewed as negative (21, 25). These cultural norms regarding aging and retirement negatively may impact nurses’ attitudes and intentions, leading to a lack of proactive behaviors to prepare for old age (8, 25). Particularly, nurses’ job stress can be increased not only by emotional labor (27, 28) but also by various structural factors (e.g., staffing ratios, shift length) (25, 29). Factors such as staffing shortages, extended work hours, inadequate promotion or welfare services within the organization, and a negative organizational culture may be all contribute to increased job stress (25, 27, 29). Such job stress may weaken the preparation for old age and health beliefs of nurses in their 20s and 30s (25, 29).

In this study, nurses caring for old adults had levels of preparation for old age and health beliefs that were mostly around or above the median. On the other hand, the mean values for financial and physical preparation, which fall into the sub-areas of preparation for old age, were slightly lower than the median. This study was based on young nurses in their 20s and 30s. It is also reasonable to assume that nurses who believe they still have sufficient time to retire prioritizing emotional preparation above financial or physical preparation in their old age planning. Specifically, it can be inferred that people are filling their lives with emotional preparation such as hobbies, self-development, and friendships as a means to meet the satisfaction of living in the moment, rather than focusing on living costs or health care (30). Based on this, it is necessary to re-identify not only young nurses in their 20s and 30s caring for old adults but also nurses caring for old adults in general by age. This needs to split their financial, physical, and emotional, preparation, and lead to pursuing further studies that consider individual and environmental characteristics.

Next, significant differences in preparation for old age were identified based on total clinical experience and level of preparation for retirement. Health beliefs also significantly differed with preparation for retirement. Those with more than 15 years of clinical experience and those with moderate or more active preparation for retirement were more likely to report higher levels of preparation for old age. Also, those who had some level of preparation for retirement had the highest levels of health beliefs. This suggests that greater clinical experience and greater preparation for retirement are associated with greater interest in retirement, which in turn leads to higher levels of preparation for old age. Also, those who had some level of preparation for retirement may be associated with higher health beliefs. Based on previous research (31) indicating that old-age preparedness is influenced by various factors, it is necessary to examine multidimensional influencing factors. Furthermore, comprehensive analysis is needed to develop effective national policies and practical measures to prepare for a super-aged society.

This study discovered that the higher the preparation for old age of nurses caring for old adults, the stronger their health beliefs. Particularly, the higher their preparation for old age, the greater their perceived sensitivity and perceived disability among their health beliefs. These findings corroborate the findings of Ha and Lee (32) who found that increased age-related health beliefs in middle-aged adults positively impact aging perceptions and preparation for old age. This demonstrates that the stronger the subjective health beliefs of nurses caring for old adults, the more likely they are to modify their behaviors to maintain and promote their health. Nurses caring for the older adults need to practice planned behaviors, such as regular health checkups and follow-up care, by strengthening health beliefs to lead more successful lives after retirement. It is necessary to utilize health care education programs that can actively practice this (32). Furthermore, additional studies are needed to identify various factors connected to preparation for old age other than the health beliefs of nurses caring for old adults.

Finally, factors influencing the preparation for old age of nurses in their 20s and 30s caring for the old adults, in order of effect size, were preparation level for retirement, department, health beliefs, desired retirement age, annual salary, and chronic illness. This suggests that preparation level for retirement, health beliefs, socioeconomic status, and health status were significant influencing factors. Also, this study found that higher annual salary and desired retirement age were associated with preparation for old age, suggesting that socioeconomic status plays a role in planning preparation for old age (31). However, while more clinical experience was associated with higher levels of preparation for old age, the impact was not statistically significant. This suggests that higher annual salary, desired retirement age, and clinical experience lead to increased consideration of retirement and, consequently, increased interest in preparation for old age. These results support the qualitative study by Nimrod and Ben-Shem (33), which aimed to elucidate the meaning of successful old-age preparation through the life journeys of older adults aged 65–92. Starting from a young age, practicing various health behaviors related to health beliefs, such as regular physical activity, nutrition, health checkups, retirement preparation according to socioeconomic status, financial ability, religion, and hobbies, is the most important factor in successfully preparing for old age. This finding supports Icek Ajzen’s Theory of Planned Behavior (TPB) (18). Behavioral intentions, shaped by three factors: attitude toward behavior, subjective norms, and perceived behavioral control, drive individuals’ planned behaviors. Health beliefs can be viewed as these behavioral intentions, leading to planned behaviors like preparation for old age. From a life course perspective (19), this study examines how early experiences can impact later life. This study suggests that young nurses in their 20s and 30s’ early experiences caring for the old adults can influence their preparation for the old age, the last period they experience in life. Therefore, maintaining a healthy lifestyle based on health beliefs is not only essential for maintaining current health, but can also enhance preparation for old age, the final stage of life, and serve as a foundation for positive outcomes in maintaining holistic health in old age after retirement.

### Implications for practice, policy, and research

Based on the results from this study, it is essential to utilize various educational programs with strengthening their health belief targeting nurses in their 20s and 30s so they can practice healthy behaviors in their daily lives, in addition to the expected behaviors in medical settings and public places. Furthermore, active participation and efforts from national and local communities are essential. Above all, there is a need for better education and training on health beliefs to keep nurses caring for old adults aware of their perceived sensitivity and motivation for preparation for old age (34, 35). To prepare for old age, it is necessary to reinforce education on health awareness, health maintenance, and disease prevention behaviors from the pre-old age stage. It is also important to develop and implement specific nursing intervention programs that can promote the implementation of health practices (32, 35). Based on research findings, we need to further examine in detail the preparation for old age of nurses caring for old adults by reflecting on South Korea’s characteristics to prepare for a super-aged society. In further study, we need to conduct a study comparing the preparation for old age of nurses per nursing department with the preparation for old age of other occupations. In particular, we propose in-depth qualitative research that presents positive causes and situations affecting the preparation for old age of nurses caring for old adults, as well as environmental factors and personal attributes.

## Limitations

The above study’s findings are significant as they provide invaluable insights into the impact on preparation for old age of nurses caring for old adults through the correlation of their health beliefs, focusing on young nurses in their 20s and 30s in South Korea. However, excluding nurse managers may eliminate valuable insights from those with broader healthcare and aging-related experience. Since the subjects of this study were young nurses in their 20s and 30s caring for old adults only in general hospitals in South Korea, it is difficult to generalize the results of this study as the impacts on the preparation for old age of nurses all in their 20s and 30s caring for old adults in South Korea as a whole. Also, the data collection window (October to November 2023) was relatively short and may miss temporal variations or seasonal influences. Data were collected only from nurses, ignoring patient feedback or institutional policies that may influence behavior. Furthermore, nurses might alter their responses simply because they knew they were being studied. They may be limitations of this study.

## Conclusion

In conclusion, the level of preparation for retirement was the most influential on the preparation for old age of young nurses in their 20s and 30s caring for old adults, followed by department, health beliefs, desired retirement age, annual salary, and chronic diseases. Based on this finding, it may be necessary to induce a positive shift in the perception of young nurses in their 20s and 30s caring for old adults to prepare for life after retirement. Additionally, it is imperative to identify and examine various potential predictors of preparation for old age for young nurses caring for old adults. The trend toward an aging society is rapidly increasing the number of old adults; hence, the scope of work of nurses caring for old adults is continuously expanding. Considering such circumstances, the current study on preparation for old age for nurses caring for old adults may be significant and valuable.

## Data Availability

The original contributions presented in the study are included in the article/supplementary material, further inquiries can be directed to the corresponding author.
